# Affect-Logic, Embodiment, Synergetics, and the Free Energy Principle: New Approaches to the Understanding and Treatment of Schizophrenia

**DOI:** 10.3390/e23121619

**Published:** 2021-12-01

**Authors:** Luc Ciompi, Wolfgang Tschacher

**Affiliations:** 1 Formerly Medical Director of the Social-Psychiatric University Hospital, CH-3010 Bern, Switzerland; lucciompi57@gmail.com; 2 Department of Experimental Psychology, University Hospital of Psychiatry and Psychotherapy, University of Bern, CH-3060 Bern, Switzerland

**Keywords:** schizophrenia, phase transition, emotions, embodiment, self-organization

## Abstract

This theoretical paper explores the affect-logic approach to schizophrenia in light of the general complexity theories of cognition: embodied cognition, Haken’s synergetics, and Friston’s free energy principle. According to affect-logic, the mental apparatus is an embodied system open to its environment, driven by bioenergetic inputs of emotions. Emotions are rooted in goal-directed embodied states selected by evolutionary pressure for coping with specific situations such as fight, flight, attachment, and others. According to synergetics, nonlinear bifurcations and the emergence of new global patterns occur in open systems when control parameters reach a critical level. Applied to the emergence of psychotic states, synergetics and the proposed energetic understanding of emotions lead to the hypothesis that critical levels of emotional tension may be responsible for the transition from normal to psychotic modes of functioning in vulnerable individuals. In addition, the free energy principle through learning suggests that psychotic symptoms correspond to alternative modes of minimizing free energy, which then entails distorted perceptions of the body, self, and reality. This synthetic formulation has implications for novel therapeutic and preventive strategies in the treatment of psychoses, among these are milieu-therapeutic approaches of the Soteria type that focus on a sustained reduction of emotional tension and phenomenologically oriented methods for improving the perception of body, self, and reality.

## 1. Introduction

During recent decades, several comprehensive theoretical accounts have emerged that are potentially relevant for a better understanding of schizophrenia, among them are the concepts of affect-logic [1,2,3], embodiment [4], synergetics [5,6], and the free energy principle [7]. These theories were developed largely independent of each other and remained only loosely connected in spite of interesting commonalities. The goal of the present paper is to explore shared features of these four concepts by focusing on common or complementary elements with the hope of identifying novel therapeutic approaches to schizophrenia.

Affect-logic is a comprehensive metatheory of cognition that focuses on the interactions between emotions (affectivity) and cognition (logic). Affect-logic is rooted in psychology, psychiatry, neurobiology, and evolutionary theory, adopting a systems-theoretical perspective, including the theory of nonlinear dynamics in complex open systems. The original German term, Affektlogik, points to omnipresent circular interactions between emotion and cognition, where affect (Latin afficere, i.e., “arousing”, “attuning”) is understood as a global bioenergetic and psychophysical state of varying quality, duration, and degree of awareness. Here, affect is an umbrella term that comprises all variants of overlapping emotion-near phenomena such as feelings, sensations, or moods. Affective and cognitive elements interact in all mental processes, where processes seemingly characterized by neutrality or indifference are likewise affective states in the sense mentioned. Simultaneously active emotions, cognitions, and behaviors are memorized as integrated “programs” for feeling, thinking, and behaving (FTB-programs). FTB-programs are learned schemas that form the essential building blocks of the psyche and are reactivated in similar situations. Conscious and unconscious emotions related to past or present experiences guide and connect all cognitive functions, such as perception, attention, memory, thought, and decision-making. In this, affect-logic concurs with Damasio’s somatic marker hypothesis that cognitive decisions are guided by emotions [8]. Emotions have focusing, selecting, and filtering “operator effects” on cognition. They tend to focus on cognitions with similar emotional tuning and to ignore cognitions with dissimilar tuning. Initially intense conscious emotions related to new, exciting, difficult, or potentially dangerous situations gradually become automated and largely unconscious yet may still continue to exert their operator effects on cognition, including in seemingly non-emotional situations. This is true even in scientific or mathematical activities, where initially intense “eureka-feelings”, which have accompanied a new discovery or solution, may gradually turn into easy-going “highways” for semi-automatized mental operations.

One aspect of emotion is, as we will see below, of particular importance for the dynamics of psychosis: their energetic aspect. Emotional phenomena are rooted in embodied bioenergetic states, which drive and motivate, sometimes also block and freeze, all motor and cognitive behaviors. These states were selected by evolution to cope adequately with situations relevant to survival, such as exploration, fight, flight, attachment, or loss, which are eventually experienced as curiosity, rage, fear, pleasure, or mourning [9]. The notion of “energy” is not just a metaphor but corresponds to the measurable allocation of energy in the form of sympathetic/parasympathetic activations of the organism in specific situations. In terms of systems theory, emotional energies provide the dynamics (the “fuel”), whereas cognitive distinctions provide the structure (the “channels”) for all kinds of mental and social systems and activities.

**Figure 1:**
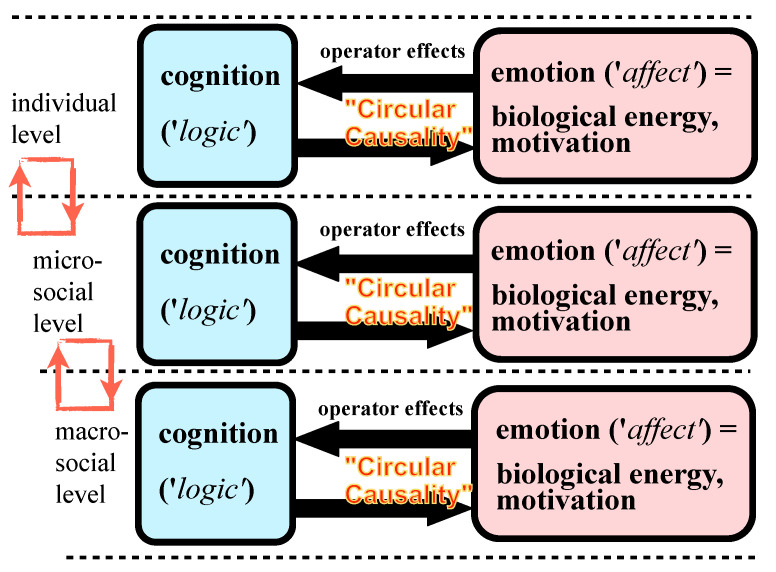
Affect-logic: cognition and emotion interact in a circular way on individual, microsocial, and macrosocial levels. Levels are linked by structural coupling. Cognitions provide the structure, whereas emotions provide the dynamics (energy, motivation) of complex mental and social systems.

The concept of affect-logic postulates, furthermore, that affective-cognitive interactions have a scale-free (so-called fractal or self-similar) architecture, as they are formally similar on individual, microsocial, and macrosocial levels [10]. The levels interact through structural coupling [11]. Self-similar selecting and filtering operator effects of emotions on attention, perception, memory, and thought act on various individual or collective levels (Figure 1), in local and short-term as well as in extended and long-lasting mental and social processes [12,13].

The affect-logic approach was developed and elaborated to serve as a metatheoretical background, especially for a better understanding of the outbreak of psychosis in the context of schizophrenia spectrum disorder. In the present theoretical overview, we wish to compare the affect-logic approach with further theoretical ‘approaches’ in the sense of encompassing theories and paradigms of cognition. We focused on systems approaches that aim at modelling the temporal dynamics of mental processes as a complex system. Three general dynamical-systems approaches were identified: embodied cognition (recently extended to 4E cognition: [14]), Haken’s synergetics, and Friston’s free energy principle. These theories, described in the following, have remained only loosely connected with each other in spite of apparent commonalities. In the following, we will explore their commonalities to derive implications and novel perspectives for the modelling of psychosis in schizophrenia-spectrum disorder and arrive at inspirations for treatment.

## 2. Embodied Cognition

**Figure 2:**
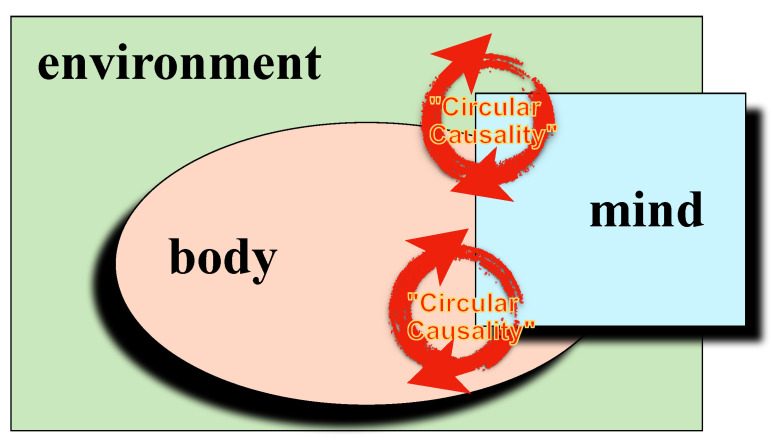
Embodiment: the mind is anchored in the body (lower loop: embodied). Body and mind are embedded in the environment (upper loop: enactive) and interact circularly in all mental activities.

The core tenet of embodiment is that mental processes, including feelings and thoughts, are anchored in the body, whether through the release of hormones, neural activity, or through behaviors (“body language”). In the process of communication between two (or more) persons, non-verbal synchronies occur regularly, as for example in the unconscious mimicry of gestures, postures, tone of voice, and facial expressions of people we interact with. Interacting individuals also tend to synchronize their physiological processes, such as skin conductance, respiration, or heart rate [15]. A characteristic of embodiment is bidirectionality, which emphasizes that the connections between mind and body go in both directions (Figure 2). In terms of the broad concept of 4E cognition, we focus here on just 2 E’s, *E*mbodied and *E*nactive. Enactivism [16] encompasses mind, body and the environment; cognition is to be understood as the active continuous interplay between sensory perception (“environment”), the contingent adaptation of motor behavior (“body”), and cognitive models of the environment (“mind”). This is symbolized by the upper loop in Figure 2. Such enactive sensory-motor loops are the basis of phenomena of nonverbal synchrony in social interaction and psychotherapy, specifically [17].

An example for the *E*mbodied loop is as follows: We smile when we feel joy, but joy can also be induced by the activation of smile-related facial muscles under a pretext. In the first case, the emotion is expressed in the body, while in the second the body expresses itself, so to speak, in the perceived emotion. For instance, the manipulation of the motor system by a “depressive” or “happy” gait on a conveyor belt correspondingly changes mental processes and influences the memory of previously learned words. Depressive walkers were found to show a bias in favor of recalling words with negative emotional content [18].

In schizophrenia, sensorimotor and physiological synchronizations are often deficient or completely absent [19]. This phenomenon, called disembodiment, may also be the source of a number of disordered bodily sensations commonly observed in schizophrenia. Such symptoms have been extensively described as so-called basic schizophrenic disorders by Süllwold [20] and Huber [21]. According to current phenomenological research, many of these disturbing body sensations may be at the origin of the distorted perception of reality and self [22,23,24]. Other mainly somatic manifestations of psychosis, such as catatonia, psychomotor agitation, or mutism-negativism, are closely related to the phenomenon of disembodiment.

The concepts and fields of embodiment/disembodiment and affect-logic are largely overlapping. The first and main reason for their correspondence is that, according to affect-logic, all emotional phenomena are somatically rooted and thus embodied. Secondly, the synchronies studied by embodiment research are crucial for the emergence of emotional contagion [25] and, hence, for the mentioned self-similar effects of emotions at the microsocial and macrosocial levels as proposed by affect-logic.

## 3. Synergetics

**Figure 3:**
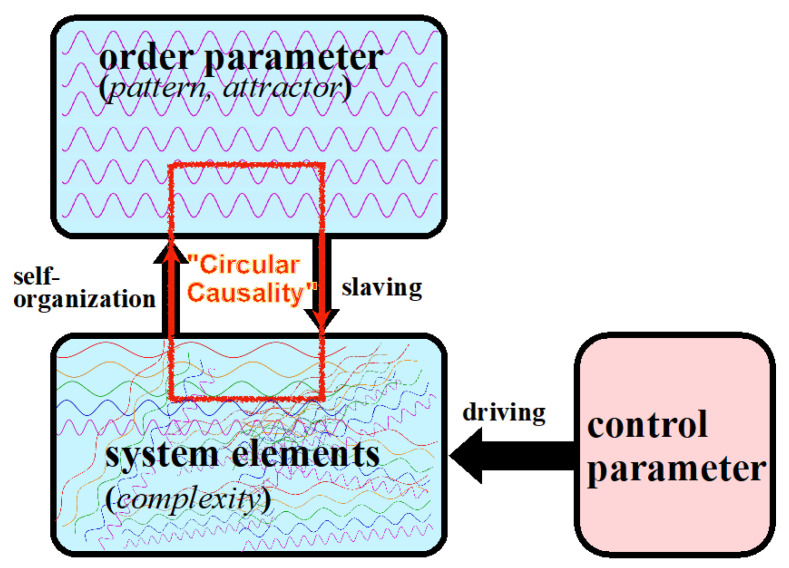
Synergetics: the complex patterns of self-organizing systems, symbolized by disordered wave lines, are synchronized, and coordinated by structural elements, the order parameters. These patterns can be disturbed and globally altered by critically increasing energetic tensions, when the input of energy (=control parameter) reaches a critical level.

The concepts that underwrite synergetics were developed in the context of the dynamics of complex open systems (or complexity theory) by the physicist Hermann Haken [5,6,26]. Synergetics is an interdisciplinary theory that models processes and mechanisms of pattern formation in physical and chemical, but also biological, mental, and social systems. Spontaneous pattern formation through self-organization depends on a system being driven by external energy sources (hence “open” system). The pattern that arises has dynamical stability and thus can be described as an “attractor”. A core phenomenon of synergetics is that sudden nonlinear bifurcations from one global pattern of functioning into another, corresponding to a transition from one basin of attraction to another, occur in complex systems of different kinds when the input of energy to the system reaches a critical level [27,28]; for visual illustrations [29]. In the terminology of synergetics, the energy input acts as a control parameter that determines the moment of bifurcation. The new pattern of functioning is shaped by a new so-called order parameter (or “nucleus of crystallization”) around which the new functional dynamic is organized. This order parameter is often a formerly peripheral structural element that suddenly becomes dominant and “enslaves” the dynamics of the whole system (Figure 3). An example from physics is the sudden transition of a random mixture of light waves into a highly ordered laser beam at a certain critical threshold of the energy supply.

Examples from the biological domain include the change of a horse’s gait from trot to gallop (both are attractors of movement coordination) under increasing energetic stimulation. In the psychosocial context, it is a common observation that critically increasing emotional tensions can provoke sudden shifts between one global pattern of feeling, thinking, and behaving into another. Thus, an initially merely verbal argument may turn into a raging brawl, a diffuse fear into a collective panic, a festering conflict into open warfare. Tschacher and Haken [30] have also applied synergetic modelling approaches to psychotherapy, distinguishing between different types of intervention in the framework of the Fokker–Planck equation (FPE). The FPE defines the change of the probability of a state variable *x* depending on time *t*. In the simplest case, *x*(*t*) is represented by Gaussian normal distribution. The probability of this distribution can be changed by a deterministic “drift” term of the equation and a stochastic “diffusion” term, hence by causation and/or chance: “change = causation + chance”. Causation can shift the location of the Gaussian distribution, whereas the stochastic diffusion term of the FPE (chance) can be expressed by changing the variance of the Gaussian. The FPE combines both dynamics and thus integrates causation with chance in a change model.

Considering the energetic properties of emotions under affect theory, we hypothesize that such bifurcations are also at work during the emergence of psychotic symptomatology. Emotional tensions can overburden a vulnerable coping system and enforce a transition from normal patterns of feeling, thinking, and behaving into psychotic patterns. The level of emotional tension is the relevant control parameter here. This tension can turn a formerly marginal structural element, such as a vague suspicion or odd behavior, into the new order parameter around which a psychotic pattern (e.g., a structured system of persecutory delusions) emerges. This hypothesis is supported both by classical clinical observations during psychotic breaks [31,32] and by the research on so-called high-expressed emotions, which has shown significant associations between the outbreak of psychosis and excessive emotional tensions in and around individuals at risk [33,34]. Emotional tensions related to traumatic experiences such as sexual abuse, migration, painful separations, or other unfavorable life events, can contribute to the progressive destabilization of a genetically vulnerable “premorbid terrain” [1,3]. In addition, they also play a crucial role in the occurrence of acute relapses during chronic long-term evolution dominated by negative symptoms [35,36].

## 4. Free Energy Principle

**Figure 4:**
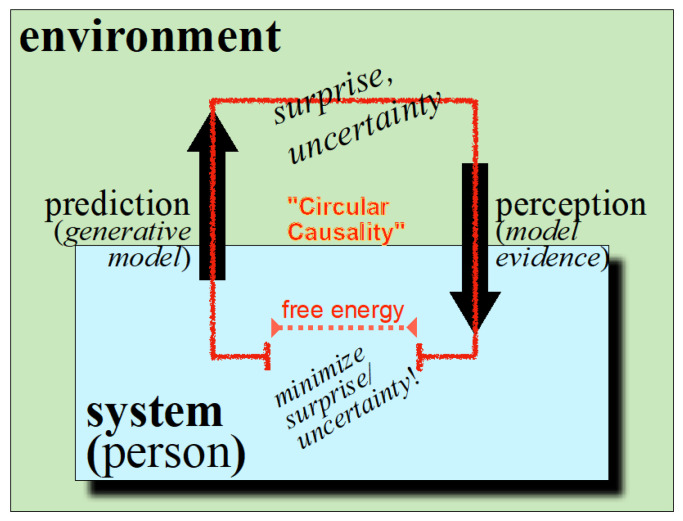
Free energy principle: biological systems continuously work on the discrepancies between predictions and perceptions, corresponding to free energy. Thus, minimizing free energy optimizes their fit with the environment, reducing surprise and uncertainty.

The free-energy principle was proposed by the neuroscientist Karl Friston. He postulated that self-organizing biological systems have the fundamental tendency of continually striving for an equilibrated energy flow with minimal losses of free energy in the service of autopoiesis [7]. In terms of systems theory, this corresponds to the minimum of the potential of an attractor, and in everyday language to the smoothest possible functioning without unpleasant surprises (where surprises can be read as states that are not part of the attracting set). Not only the single neuron but also the brain and the organism as a whole are understood as devices for improving predictions regarding the behavior of their respective environment. Mathematically, the free energy in question here stands in as an upper boundary on the likelihood of states a particular system encounters and plays the role of a potential function. Or in everyday language: free energy arises when the predictions made by the system are wrong. However, unlike thermodynamic free energy that is related to heat, the free energy in question here is a function of sensations and (Bayesian) beliefs about the causes of those sensations. Free energy minimization in biological systems operates by improving the prediction of environmental reactions on the basis of experience (Figure 4). It can then be construed as “active inference”, in which both action and perception try to minimize “surprise” (also known as a prediction error). This is equivalent to maximizing the marginal likelihood (i.e., the goodness of fit) of sensory inputs (also known as model evidence) under a generative model that is embodied by the system in question. In this setting, a prediction (i.e., generative model) generates predicted sensory consequences from inferred causes (i.e., model evidence).

Improved predictions are achieved by actively adapting the implicit model of the real world through structural and functional changes—from fast changes in neuronal activity through to the slow growth of new connections in the neuronal network—and through acting on the environment itself (e.g., by simply moving one’s eyes, or by moving one’s entire body to a more appropriate environment). Any movement changes both what is perceived and what can be predicted, thus influencing the generative models and the model evidence with the goal of improving adaptation to the environment. Expressed in statistical terms, the organism strives to maximize the likelihood of its continuous modelling loops so as to minimize free energy. According to Friston, the minimization of free energy underlies many biological phenomena. A striking example is the emergence of rhythmic oscillations in the brain known as alpha, beta, and gamma rhythms in the EEG [37,38]. All learning processes, too, can be cast as the minimization of free energy.

Friston’s ideas have predecessors in the reafference principle [39] and are also reminiscent of the interplay between assimilation and accommodation during cognitive development described by Jean Piaget [40]. Konrad Lorenz similarly postulated that every development of life is in itself equivalent to an accumulation of knowledge about the surrounding world [41]. The free energy principle shares the fundaments of affect-logic, embodiment, and synergetic formulations: For example, all rest upon a circular causality that lies at the heart of synergetics. In the case of the free energy principle, it is minimizing surprise or prediction errors to attune an individual’s implicit model of the world (the generative model) with the embodied world that supplies the sensory evidence for that model. The free energy principle is quintessentially “embodied” in the sense that the generative model is embodied or entailed by both the brain and body. Mathematically, the free energy formulation rests upon the solution to the Fokker–Planck equation and on the very existence of a random dynamical attractor, where “random” pertains to the stochastic term of the equation and “attractor” to its deterministic term. This attractor is constituted by states which the organism is actively striving to attain in the context of exogenous (deterministic) forces and (random) fluctuations that endow nonequilibrium steady states with the dynamical itinerancy evinced by bifurcations and phase transitions. Finally, the existential imperative of minimizing surprise and uncertainty speaks directly to the affective nature of self-organized, autopoietic behavior and the dynamic role of excessive emotional tensions featured in affect-logic theory.

Friston’s concepts complete and deepen the approaches of affect-logic and synergetics in an interesting way. They suggest, in particular, that the anxiety, insecurity, and critical emotional tension, which usually precede the outbreak of psychosis, may represent a clinical manifestation of increased free energy (i.e., ego-dystonic unpredictability and uncertainty). The production and perception of psychotic symptoms such as delusions, hallucinations, catatonia etc. may thus correspond to attempts of the mental apparatus to control and minimize free energy through the creation of an “alternative reality” in the service of autopoiesis. This interpretation is supported by clinical observations, which show that the emergence of a coherent system of delusions, or of negative psychotic symptoms such as indifference and social isolation, is often accompanied by a decrease of overt emotional tension and anxiety [31,32]. The inference aspect of active inference manifests clearly in this reading of the free energy principle in terms of false inference: namely, inferring things are there when they are not (e.g., hallucinations and delusions) or inferring things are not there when they are (e.g., dissociative symptoms, derealization, depersonalization).

Tschacher, Giersch, and Friston [19] assume that psychotic interpretations of reality are based on erroneous predictions possibly related to disturbed perceptions of the environment and, in particular, the embodied self. Erroneous predictions may also be the consequence and, simultaneously, the cause of the “loss of normal self-evidence” and disembodiment emphasized by phenomenologists such as Blankenburg and Fuchs . The blurred boundaries between self and others focused on by psychoanalysts [42] point in the same direction. Similarly, unfamiliar environments and conflicting or contradictory life experiences (“double-binds”), which were found to be related to the outbreak of psychosis [43,44], may contribute to false inferences and ensuing (erroneous) predictions.

## 5. Discussion: Towards a Translational Understanding of Psychosis

A translational, that is to say, multi-conceptual, understanding of schizophrenia arises on the basis of the above, and this translational view can be integrated into the generally accepted vulnerability–stress model of psychiatry, which postulates that acute psychotic symptoms can arise in both genetically and/or biographically vulnerable individuals when emotional tensions induced by stress—surprise or uncertainty—reach a critical level [1,45,46,47]. These tensions correspond to a clinical manifestation of unbound free energy, which is eventually minimized by a nonlinear phase transition from normal to psychotic patterns of feeling, thinking, and behaving.

In synergetics and the free energy approach, the dynamics of phase transitions is viewed as the process of moving through bifurcations. In other words, the currently active attractor becomes unstable and increasingly weakened before the critical point of the control parameter or free energy is reached [6]. The signature of this destabilization of the old pattern/attractor is called critical slowing down, which can be observed empirically as the system needs more time to relax into its attractor after some perturbation. At the exact point of bifurcation, the system “chooses” between two (new) quasi-attractors, which choice often occurs through a chance event. This means, however, that exactly this period in the development of the system is most accessible for (even small) deterministic interventions that can guide the trajectory into the preferred specific attractor.

This innovative view has the advantage of not only integrating the four theoretical approaches in question. It also better explains the enormous variability of long-term outcomes revealed by follow-up studies over several decades [48,49,50,51]. Furthermore, psychotic symptoms such as delusions, hallucinations, emotional indifference, and social withdrawal do not appear, in this light, merely as deficits in the sense of the standard medical model but also as active and productive coping strategies. Symptoms are thus understood as instrumental in the service of free energy minimization and autopoiesis, as symptoms decrease the critical emotional tensions. Over time, these defensive mechanisms may become ingrained, habitual, and hard-wired by neural plasticity—thus partly explaining certain functional and structural modifications found in the brains of persons with schizophrenia [52,53].

## 6. Therapeutic Implications

The proposed understanding of schizophrenia in the context of complexity science has both therapeutic and preventive implications. We consider the following of particular interest:

We noted above that, according to complexity theory, the critical time of phase transitions are particularly sensitive for interventions. The critical slowing down of a system, the signature of being close to imminent bifurcation, is in principle clinically observable and can thus inform about the optimal time for deterministic interventions towards desired new cognition or behavior.

If excessive emotional tensions do indeed play the postulated key role at the onset of psychosis, then systematically reducing emotional tensions in and around acute patients should be a main therapeutic focus. Many elements of conventional medical interventions, however, rather increase than decrease emotional tensions, among them the often chaotic circumstances of hospitalization, the unfamiliar and opaque atmosphere of psychiatric hospitals, size of wards, architecture, lack of staff continuity, overstimulation by excessive noise, and sometimes also violence. Small, family-like, stimulus-protecting environments should therefore be much more appropriate. In terms of the Fokker–Planck Equation (FPE), such interventions relate to the stochastic term of the FPE. Stochastic interventions can be implemented to protect existing patterns from random fluctuations by boundary regulation [30].

This is realized in therapeutic environments of the Soteria type, which were first created in the 1970s in California [54] and have been functioning successfully since 1984, with some conceptual modifications, in the therapeutic community Soteria Bern in Switzerland [55,56]. An increasing number of similar institutions were recently founded across Europe, especially in Germany. In this approach, sustained emotional relaxation is not mainly obtained by neuroleptic medication, but rather by continual personal support, by “being with” and “doing with” the psychotic patient, creating a trusting therapeutic alliance between specially selected and trained caregivers and the patient. The Soteria milieu is designed to ward off overtaxing external inputs when the patient initially lives in the “soft room”, which corresponds to boundary regulation. Further ingredients of the treatment philosophy are the systematic inclusion of the patient’s family or other important persons of reference into the therapeutic process and participation in appropriately dosed everyday activities such as cooking, shopping, and housekeeping. According to comparative empirical research, two-year results were obtained that are equivalent to, and subjectively rather better than, standard treatment. Soteria-type environments use significantly fewer neuroleptics and can partly also be run with lower costs [56,57,58,59].

A stable, transparent, and securing therapeutic environment of the Soteria type may also improve patients’ predictions about the behavior of their environment and, thus, reduce the need for “crazy” alternative explanations. According to a recent pilot study conducted by Soteria Konstanz (Germany), disturbed feelings of reality and self can be significantly improved by flexibly integrating specific behavior–therapeutic elements into everyday contacts and activities [60].

Similar goals are pursued by body-centered therapeutic methods recently proposed in connection with the disembodiment in schizophrenia, such as interventions from dance and movement therapy. These approaches emphasize the significance of sensorimotor experience and body motion for cognition, affect, and social interaction and strive to enhance emotional processing and self-regulation [61,62]. Such methods appear especially effective for reducing negative symptoms such as passivity and social withdrawal [63].

Innovative therapeutic strategies may also be based on the previously mentioned productivity of psychotic symptoms, e.g., by valorizing the creative aspects of the involved emotional energies and eventually steering them towards more constructive directions. An example is Milton Erickson’s imitating the incomprehensible artificial language of a chronically psychotic patient and regularly “speaking” with him in this language until the patient suddenly started to speak normally, gradually accepted a friendly relationship and progressively normalized his behavior [64].

Finally, the multifocal understanding of psychosis proposed here strongly argues for restructuring long-term psychotherapy. Conventional long-term neuroleptic medication is not only unable to consolidate the underlying structural vulnerability of the patient but is also burdened by severe long-term side effects. Among the many currently proposed psychotherapeutic methods, the following two are particularly close to the proposed understanding of psychosis: The approach practiced by the Swiss family therapist Ursula Davatz, who describes psychosis as the result of an “emotional tsunami” in the family system [65], and the integrative approach developed by the Italian therapist Giovanni Ariano [66,67]. Since, as we believe, emotions do play a decisive role in the outbreak of psychoses, it may also be worth considering interventions developed in psychotherapies for other disorders, such as depression. For instance, it was found that emotion regulation plays a role in depressive disorders, where some patients are emotionally over-regulated whereas others are under-regulated. It is thus important to take the type of emotion regulation into account in the choice of therapeutic strategies [68], which appears reasonable also in psychotherapy with schizophrenia patients.

## 7. Open Questions

The synopsis of the concepts of affect-logic, embodiment, synergetics, and free energy minimization appears capable of opening up new ways of understanding and also treating schizophrenia. In this view, embodied emotions and emotional energies play a more important role in the genesis and long-term evolution of schizophrenia than hitherto admitted. Unbounded (“free”) emotional energies related to traumatic life experiences not only contribute to further destabilizing a vulnerable “premorbid terrain” but are probably also responsible both for the outbreak of acute psychotic decompensations, as well as for acute relapses during long-term clinical progression characterized by negative symptoms. This same view also stimulates a number of lingering open questions:

- Why do certain people react to critically rising emotional tensions with psychotic symptoms, whereas others respond with violence, fall into panic, or become depressed?
- Is there a specific schizophrenogenic vulnerability, and what does it consist of? It was long suspected by the affect-logic perspective, with special reference to Eugen Bleuler’s concept of “loose associations” (cf. disembodiment!), that there may be a partly genetically and partly biographically determined instability of the links between feeling and thinking. Of particular importance are those links that regulate basic interpersonal relations (in psychoanalytic terminology, the object representations). This hypothesis is at least partly supported by recent neurobiological findings revealing disturbed neuronal connections between frontal cortical and subcortical areas, the amygdalae [69].
- Does the labilization of the psyche that is physically manifest in terms of functional disconnections in the brain result as a direct consequence of aberrant (i.e., false) learning and inference [70,71]?
- There is yet no direct quantitative evidence for the hypothesis that emotional tensions can lead to a phase transition into psychosis, as is proposed by affect-logic. It would be desirable to have objective physiological markers for emotional tension at hand.
- Why does the psychotic phenomenology differ so much from case to case that Eugen Bleuler, the creator of the term schizophrenia in 1911, used to speak of “the group of schizophrenias” [72]? Is this related to the changing influence of a great number of environmental variables or to some genetic or other biological variables?
- As a final and perhaps most important, but to our knowledge astonishingly neglected, question: How do both “spontaneous” and therapy-induced improvements and recoveries arise? According to long-term follow-up studies over several decades [48,50,73], full and lasting recoveries comprise, in the long run, at least one fourth of cases and may even amount to roughly two thirds under especially favorable conditions [51,74].

It is obvious that all these questions and hypotheses, while quite plausible in our eyes and also consistent with a number of clinical observations and empirical findings, need much more specification and confirmation—or rejection—by further research. The goal of the present explorative (and perhaps provocative) overview was to stimulate such research.

## 8. Endnotes

1. The Fokker-Planck equation plays a central role in nearly all of physics. It describes the evolution of the probability density of a system’s states when they are subject to random fluctuations and deterministic impacts. Common variants of the Fokker-Planck equation include the master equation for discrete systems and the Schrödinger wave equation in quantum electrodynamics. It also manifests in theoretical biology as a model of population density dynamics (e.g., the Wright-Fisher model). The solution of the Fokker-Planck equation for any random dynamical system—in a nonequilibrium steady state—also forms the basis of the free energy principle.

2. Mathematically on the background of the Fokker-Planck equation, uncertainty can be read as an expected surprise. In information theory, surprise (or surprisal) corresponds to self-information, while expected surprise or uncertainty is known as entropy. Minimizing expected free energy by choosing appropriate actions can then be read as reducing uncertainty in anticipation of familiar, unsurprising outcomes. From a physicist’s perspective, this would look like self-organization to a nonequilibrium steady state. From a psychiatrist’s perspective, this would look like an active search for synchrony and predictability and the active avoidance of existential fear.

3. We use ‘unbound’ here deliberately to conflate Freudian notions of unbound energy with the role of variational free energy as a bound on surprise or marginal likelihood [75]. Indeed, in machine learning, variational free energy is known as an evidence bound [76].
